# National Hospital for the Paralysed and Epileptic

**Published:** 1903-05-09

**Authors:** 


### NATIONAL HOSPITAL FOR THE PARALYSED AND EPILEPTIC.

The Lord Mayor (Sir Marcus Samuel) presided at the festival dinner of the National Hospital for the Paralysed and Epileptic at the Hôtel Metropole on the 30th ultimo.

In proposing the toast of the evening, "Success to the Hospital," the Chairman said that in order to make himself familiar with the subject on which he was to speak he had visited the hospital, and, under the guidance of the chairman, Dr. Wace, had gone thoroughly over what he must describe as their very fine hospital—a course he would recommend to everyone, since it was most centrally situated, and thus involved no loss of time. If they did so they would certainly realise that the institution was in very many respects quite up to date. They must bear in mind what was the work which distinguished their hospital from the hundreds of other hospitals in the Kingdom. It was an attempt to elucidate and cure some of the most terrible of the evils which afflict the human race. Epilepsy was at length beginning to be understood, and it was realised that in some instances at least it was curable. Experts could tell from the way a patient moved his or her limbs what was the seat of the trouble, and the doctors were able to guide the surgeons to where the depression of the brain lay, so that they were able to effect cures by surgery which a few years ago would have been wholly impossible. But if that appealed to their pity on the one side, it also appealed to their selfishness also, because one of the great causes of paralysis was one to which they were all in these days liable—overwork and overstrain of the nervous system, and that hospital formed a school for the whole world where it was to be hoped and expected that great practical advances in the treatment of the disease would continue to be made and perfected. It was not a small work that was being done. There were 160 beds, and with the convalescent home 200 beds, so that it was the largest in the kingdom for the treatment of nervous diseases. It was founded in 1859 and for a long time carried on in old houses. In 1880 the present building was commenced, and in 1897 the convalescent home was added. Last year over 1,000 people were treated as in-patients and 6,366 as out-patients. There were also 90 pensions provided for incurable cases—an important adjunct, because, though hope was writ large on their portals, yet there were many hopeless ones, and instead of having to turn them out they had funds to enable them to alleviate the sufferings of their struggle. To do all this they had only an income of £10,000, while their expenditure reached £18,000. It was hardly fair to compare their cost with that of other hospitals because from the nature of their cases their treatment consisted largely of giving good, plentiful, and generous food; cures depending greatly upon rest and diet. Their patients were sometimes retained for as long as four months. There were some hospitals which "cooked" their statistics by discharging patients long before they should. Some kept them 18 days and some 28 days, and in comparing the number of patients dealt with he thought the credit should rest with the hospital that kept them longest. The National Hospital was only able to take the most pressing cases owing to want of funds and of space. It was badly in need of extension, and under the old regime, the old committee had secured two houses adjoining the hospital, which they now appealed for help to pull down and rebuild. They wanted an isolation ward; the theatre needed enlarging, and there was grave need for further housing accommodation for their nursing staff. They had one ward on the men's and one on the women's side for paying patients, but the payments were limited to 21s. a week, which entailed a loss of 12s. a week. That was better than a loss of the whole amount, and it saved self-respecting people from feeling that they were beholden to charity. They paid the guinea cheerfully, because to go into a private nursing home they would have to pay 12 to 15 guineas—sums entirely beyond their means. He thought he had said all that need be said in favour of supporting this institution. A sum of £20,000 to £25,000 was required to enable them to utilise the ground they had and so extend their beneficent mission and relieve the managers from their anxieties.

The Very Rev. Dr. Wace responded. He thanked the Lord Mayor for the great pains he had taken to make himself acquainted with the circumstances of the hospital. He was happy to be able to say that the general state of the work and the finances was satisfactory. The late board had administered these in a very admirable manner. But the first point to be looked to in the administration of a hospital was, that all branches should be working perfectly harmoniously together, and that they were now doing. He regretted that from that day other duties would take him from an association in which he had taken the greatest interest, but he should always be ready to render them all the assistance in his power. (Cheers.)

Sir John Cockburn, who proposed the health of the medical and surgical staff, endorsed every word that had been said as to the extent to which the influence of that hospital pervaded the British Dominions. It was a school for the whole world.

Sir William Gowers replied for the staff. As to the influence of the institution outside, they had some 500 doctors studying there every year and then returning to practice; this had been going on for twenty years. But however highly they stood as a school, the well-being of their patients was always their first consideration, and nothing in the nature of experiments were ever performed on them.

The amount collected was announced as £1,882, and Mr. Justice Kennedy having proposed the Chairman's health the proceedings were brought to a close.

